# The Translation Elongation Factor eEF-1Bβ1 Is Involved in Cell Wall Biosynthesis and Plant Development in *Arabidopsis thaliana*

**DOI:** 10.1371/journal.pone.0030425

**Published:** 2012-01-17

**Authors:** Zakir Hossain, Lisa Amyot, Brian McGarvey, Margaret Gruber, Jinwook Jung, Abdelali Hannoufa

**Affiliations:** 1 Agriculture and Agri-Food Canada, London, Ontario, Canada; 2 Department of Biology, University of Western Ontario, London, Ontario, Canada; 3 Agriculture and Agri-Food Canada, Saskatoon, Saskatchewan, Canada; University of Melbourne, Australia

## Abstract

The eukaryotic translation elongation factor eEF-1Bβ1 (EF1Bβ) is a guanine nucleotide exchange factor that plays an important role in translation elongation. In this study, we show that the EF1Bβ protein is localized in the plasma membrane and cytoplasm, and that the transcripts should be expressed in most tissue types in seedlings. Sectioning of the inflorescence stem revealed that EF1Bβ predominantly localizes to the xylem vessels and in the interfascicular cambium. *EF1Bβ* gene silencing in *efβ* caused a dwarf phenotype with 38% and 20% reduction in total lignin and crystalline cellulose, respectively. This loss-of-function mutant also had a lower S/G lignin monomer ratio relative to wild type plants, but no changes were detected in a gain-of-function mutant transformed with the *EF1Bβ* gene. Histochemical analysis showed a reduced vascular apparatus, including smaller xylem vessels in the inflorescence stem of the loss-of-function mutant. Over-expression of *EF1Bβ* in an *eli1* mutant background restored a WT phenotype and abolished ectopic lignin deposition as well as cell expansion defects in the mutant. Taken together, these data strongly suggest a role for EF1Bβ in plant development and cell wall formation in Arabidopsis.

## Introduction

Translation is one of the vital processes involved in the fine regulation of gene expression through ensuring direct, rapid, reversible and spatial control of protein concentration [1], and thereby affects developmental processes in both prokaryotes and eukaryotes. Translation elongation in eukaryotes requires a set of soluble non-ribosomal proteins known as eukaryotic elongation factors or eEFs [2]. They include eEF1A and eEF1B factors, which are involved in the recruitment of aminoacyl-tRNAs onto the ribosome, and eEF2 factor, which mediates ribosomal translocation. eEF1B is essential for growth [3] and plays a role in oxidative stress resistance in yeast [4]. eEF1B is also involved in distributing eEF1A between polypeptide chain elongation and actin-binding activities [5], and in cell cycle regulation [6].

The plant eEF1B is a trimer composed of the structural protein (eEF1Bγ) plus two nucleotide exchange subunits (eEF1Bα and eEF1Bβ) [6] and is intermediate in complexity between yeast and metazoans. The yeast eEF1B is made up of two subunits, a guanine nucleotide exchange protein (eEF1Bα) and a structural protein (eEF1Bγ), whereas the metazoan complex is a heteromer of at least four subunits: the structural protein (eEF1Bγ), two nucleotide exchange factors (eEF1Bα and eEF1Bδ), plus the unique valine-tRNA synthetase (Val-RS) [6]. The nucleotide exchange function is achieved primarily by the eEF1Bα isoform, and the exact physiological functions of eEF1Bβ and eEF1Bδ are not yet known.

The plant cell wall is a complex and dynamic structure composed of polysaccharides (cellulose and hemi-cellulose), proteins, and phenolic compounds (primarily lignin, but also other phenolic acid linkages) [7]. The cell wall not only strengthens the plant body, but also plays key roles in plant growth, cell differentiation, intercellular communication, water movement and defense [8]. Disruption of either cellulose or lignin biosynthetic and regulatory genes leads to stunted phenotypes, irregular xylem development and weak stem formation [9], [10], but the link between disruption of monolignol biosynthesis and dwarfism is not clearly established [10]. Recently, a direct relationship between cell wall biosynthesis and cytoskeleton was reported [11], [12]. This was significant in light of the physical interaction established earlier between eEF1β and actin in the cytoskeleton of *Dictyostelium discoideum* [13].

In this study, we investigated the role of *eEF-1Bβ1* (locus At1g30230, referred to hereafter as *EF1Bβ*) in plant development by studying changes in cell wall structure and composition within a dwarfed T-DNA insertion line, SALK_046102C [14]; referred to hereafter as *efβ*, as a result of the mis-expression of the *EF1Bβ* gene. We also investigated the impact of *EF1Bβ* over-expression in the Arabidopsis *ectopic lignification 1* (allele *eli1-1*, hereafter *eli1*) mutant. This plant line has a mutation within cellulose synthase CESA3 and exhibits ectopic lignification in cells normally free of lignin [15]. Using these genetic tools, we show that EF1Bβ not only affects plant growth and cell elongation, but also plays a role in the biosynthesis of cellulose and lignin and points to EF1Bβ as a novel regulator of plant development and cell wall biosynthesis.

## Materials and Methods

### Plant materials and growth conditions

SALK_046102C seeds were obtained from the Arabidopsis Biological Resource Center (ABRC), Ohio State University, USA [14]. Plants from this accession were mutated in the *EF1Bβ* gene (At1g30230) and did not show any phenotype until the following generation, when we observed 11 plants (*efβ*) with strong dwarf phenotypes out of 40 plants, indicating that the original SALK_046102C line was heterozygous. The *eli1* mutant line was obtained from Dr. D. Bonetta at the University of Ontario Institute of Technology, Oshawa, Canada. *Arabidopsis thaliana*, ecotype Columbia (Col 0), and mutant lines *eli1* [15] and *efβ* were grown in Promix BX (www.premierhort.com) in a growth room (16-h light/8-h dark) under fluorescent white light (150 µmol m^−2^ s^−1^) at 22°C after stratification at 4°C for 48 hr to synchronize germination. For plate-grown seedlings, sterilized seeds were sown on 0.5× MS medium with 1% sucrose in the light, or without sucrose in the darkness. For growth in the dark, seeds were exposed to fluorescent white light (150 µmol m^−2^ s^−1^) at 22°C for 6 hr to induce germination, after which the plates were wrapped individually with aluminum foil. The age of the seedlings was defined starting at the end of the cold treatment.

### Mutant genotyping

Segregating plants from SALK_046102C seeds were used for T-DNA analysis and to develop the homozygous line, *efβ*. The homozygous dwarf plants were used in all subsequent analysis. The T-DNA insertion into the *EF1Bβ* gene was confirmed in *efβ* plants by PCR with T-DNA border and gene-specific primers (LBb1.3, LP and RP; Table S1) designed by SIGnAL T-DNA Verification Primer Design Tool (Salk Institute Genomic Analysis Laboratory, CA, USA). To determine the nature of the mutation and T-DNA copy number, the mutant was backcrossed to the WT (Col 0) and the presence of T-DNA in BC_1_F_1_ plants was confirmed by PCR. BC_1_F_2_ seeds were obtained from BC_1_F_1_ plants through selfing. BC_1_F_2_ seeds from four individual plants were grown for characterization of the *efβ* mutant. Genotyping of the *eli1* mutant was carried out previously by others [15], [16].

### Plasmid construction and transformation

The promoter and 5′-UTR of *EF1Bβ* (1931-bp fragment including the start codon ATG) was amplified from Arabidopsis genomic DNA by PCR (primers P1+P2, Table S1) and cloned into the Gateway entry vector pENTR/D-TOPO (Invitrogen). The promoter was recombined into the Gateway destination vector pMDC163 containing *UidA* gene [17] through an LR clonase reaction (Invitrogen). The coding region of *EF1Bβ* was amplified by PCR (primers P3+P4, Table S1) from Arabidopsis cDNA and cloned into pGEM-T Easy vector (Promega), then transferred into the binary vector pBINPLUS-35S as a BamHI and SacI fragment to generate the over-expression construct EFβOX. To generate a translational fusion, *EF1Bβ* cDNA without the stop codon was amplified by PCR (primers P5+P6, Table S1) from Arabidopsis leaf cDNA, cloned into pENTR/D-TOPO; then the insert was transferred into the pEarleyGate 101 vector upstream of the yellow fluorescence protein (YFP) [18] using the Gateway recombination system (Invitrogen) to create the 35S-EF1Bβ-YFP construct. All clones were confirmed by sequencing.

The constructed vectors were electroporated into *Agrobacterium tumefaciens* strain LBA4404 except for 35S-EF1Bβ-YFP, which was introduced into GV3101. Arabidopsis plants were transformed by the floral-dip method [19]. For the complementation study, the EFβOX construct was introduced into *efβ* and *eli1* mutant lines by the same method. Transgenic Arabidopsis seedlings were selected on growth medium containing 0.5× Murashige and Skoog salt mixture (*Phyto*Technology Laboratories, KS, USA), 1% (w/v) sucrose (pH 5.8), and 0.8% (w/v) plant agar (Sigma) supplemented with 25 mg/l hygromycin or 50 mg/l kanamycin. Plants positive for the T-DNA were further confirmed by PCR.

### Confocal microscopy

Roots of four-day-old Arabidopsis seedlings that were transformed with the EF1Bβ-YFP fusion construct were examined on a DM IRE2 inverted microscope equipped with an HCX PL APO 1.20 63X water-immersion objective. Images were collected in a 512×512 format on a TCS SP2 confocal system (Leica Microsystems) using a scanning speed of 400 Hz. YFP was visualized by exciting the samples with the 514 nm argon laser line and collecting fluorescence with an emission window set at 520-580 nm. To plasmolyze the cells, seedlings were incubated in ½ MS containing 0.75 M sorbitol for 15 minutes, and then incubated in 8 µM SynaptoRed™ Reagent (SR) (Calbiochem) for 5 minutes to stain the plasma membrane.

### β-glucuronidase (GUS) histochemical assay

For the GUS staining assay, 12-day-old seedlings grown on MS media and tissue collected from the base of the inflorescence stem from 6-week-old plants grown on soil were tested with 5-bromo-4-chloro-3-indolyl glucuronide according to Hématy *et al*. at 37°C for 4 h [20]. Stem pieces were mounted in 3% agarose, and 30 µm sections were prepared using a Leica VT1000S vibratome (www.leica.com). Seedlings and sections were visualized with Nikon SMZ 1500 and Zeiss Axioskop *2 plus* microscopes, and images were captured using a NIKON DXM 1200 digital camera.

### Histochemical staining for lignin

For phloroglucinol staining, 5-day-old dark grown seedlings or 30 µm sections from the base of the inflorescence stems were stained with 2% phloroglucinol in 95% ethanol and concentrated HCl (v/v, 2:1) for 5 min. For Mäule staining, 100 µm sections were treated for 10 min with 1% KMnO_4_ and then rinsed with water. Sections were then treated for 3 min with 10% HCl, rinsed in water, and mounted in concentrated NH_4_OH. For anatomical analysis of *eli1* and *eli1*-EFβOX plants, 30 µm sections from the base of 6-week-old inflorescence stem were treated with 0.02% aqueous solution of Toluidine Blue. All samples were observed under a light microscope and photographed using a NIKON DXM 1200 digital camera.

### Lignin content and monomeric composition analysis

Total lignin from the inflorescence stem was determined using the thioglycolic acid (TGA) method according to Brinkmann *et al*. [21] with slight modifications. Briefly, cell wall residue (CWR) was prepared by extracting the ground stem with toluene/ethanol (2∶1, v/v), 95% ethanol, and water (three times each). Extractive-free CWR was dried at 70°C overnight. Aliquots of 10 mg dried CWR (3 replicates per individual sample) were weighed into 2 ml screw cap tubes (Sarstedt) and mixed with 1.5 ml of 2 N HCl and 0.3 ml thioglycolic acid (TGA). Subsequent analysis was carried out as per Brinkmann *et al*. [21], and the relative amount of lignin was measured considering WT absorbance at 280 nm as 100%.

Lignin monomer composition was determined using thioacidolysis as described by Foster *et al*. [22], except that 10 mg of CWR was used as the starting material and all reagents were scaled up accordingly. We used an Agilent 7890 GC/5975 MSD with an HP-5MS column (Agilent, 30 m × 0.25 mm i.d., 0.25 µm film thickness) for monolignol analysis. Total ion chromatogram peaks were identified by relative retention times using tetracosane as an internal standard, as well as by determining characteristic mass spectrum ions of 299 m/z and 269 m/z for syringyl (S) and guaiacyl (G) monomers, respectively. The relative composition of the lignin components was quantified by setting the total peak area of the lignin peaks to 100%.

### Cellulose content analysis

Cellulose content was determined using a colorimetric method [23] and expressed as µg cellulose mg^−1^ DW.

### Quantitative real-time RT-PCR

For quantitative real time reverse transcription PCR (qRT-PCR) experiments, total RNA was extracted from 6-week-old inflorescence stems using TRIzol reagent (Invitrogen). RNA was treated with Turbo DNAse (www.ambion.com) to eliminate trace amounts of genomic DNA. Reverse transcription reactions were performed with Superscript™ III Reverse Transcriptase (Invitrogen) using 2.0 µg of RNA per reaction; then the cDNA was diluted 25-fold with nuclease-free water. Polymerase chain reactions were carried out in a 96-well plate in a LightCycler® 480 II (http://www.roche-applied-science.com/lightcycler) using SYBR® Green Master Mix (Roche) in a reaction volume of 20 µl. Five reference genes [adenine phosphorybosyl transferase 1 (At1g27450), elongation factor *EF1α* (At5g60390), eukaryotic initiation factor *elF4A1* (At3g13920), *UBC21* (At5g25760), and *UBQ10* (At4g05320)] were tested in the experiment, and the two most stable genes (*EF1α* and *elF4A1*) were selected for data normalization using geNorm software [24]. PCR efficiency was determined from amplification plots using the program LinRegPCR [25].

### Statistical analysis

The t-tests were performed using the STATISTIX for Windows 2.2 program (Analytical Software, Tallahassee, FL, USA).

## Results

### Sub-cellular localization of EF1Bβ protein to the plasma membrane

**Figure 1:**
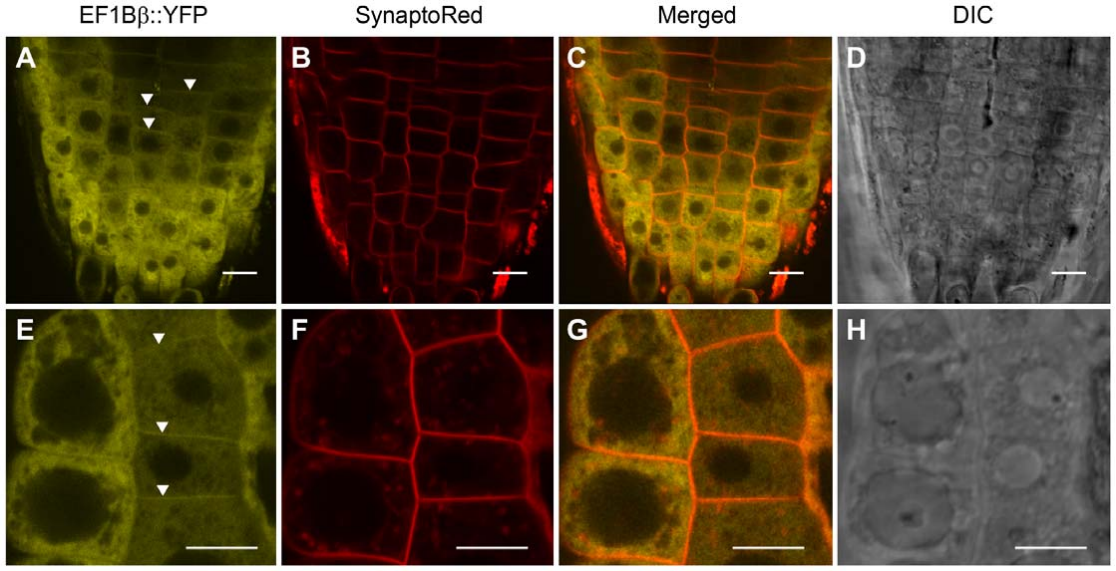
Confocal images showing localization of EF1Bβ-YFP (A and E) in Arabidopsis root tip cells plasmolyzed with 0.75 M sorbitol. SynaptoRed (SR) was used as a plasma membrane marker (B and F). Panels C and G are the merged images of the YFP and SR channels and D and H show the DIC images. Lower panels (E-H) show a close-up of root tip cells. Bars  =  10 µm. Arrowheads (A and E) highlight the YFP localization in the cell periphery.

To investigate the subcellular localization of EF1Bβ, we transformed Arabidopsis plants with a translational fusion of EF1Bβ and the yellow fluorescent protein (EF1Bβ::YFP) under the control of the 35S promoter. YFP fluorescence was observed in the cytosol and periphery of epidermal cells of the root tips of stably transformed seedlings (Figure 1A–C). Following plasmolysis with sorbitol, EF1Bβ::YFP localization remained clearly visible in the plasma membrane and cytosol (Figure 1D–F). These results are consistent with the results of proteomics studies that predicted EF1Bβ to be a plasma membrane [26], [27] as well as a cytosolic [28] protein. However, in some transformed lines, bright yellow dot like aggregates were observed in the cytoplasm after plasmolysis (Figure S1). It is possible that EF1Bβ::YFP protein partially coagulated at high expression levels because of plasmolysis although this phenomenon remains to be elucidated.

### Expression of *EF1Bβ* promoter in seedlings and inflorescence stem

**Figure 2:**
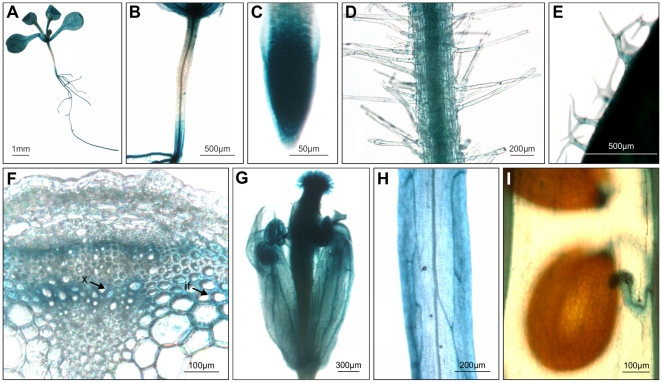
Expression pattern of *EF1Bβ* gene in Arabidopsis seedlings, inflorescence stem and reproductive organs. Transgenic Arabidopsis plants expressing *EF1Bβpromoter-UidA* reporter fusion were examined histochemically for GUS activity. Seedling (A), hypocotyl (B), root tip (C), root hairs (D), trichomes (E), cross section of inflorescence stem (F), flower (G), silique epidermis (H), and fully developed seed (I). Cross section of the stem (F) shows the expression of *EF1Bβ* in xylem vessels (x) and interfascicular fibers (if).

To investigate the expression pattern of *EF1Bβ*, 1931 bp upstream sequence containing the putative promoter region and 5′-UTR of *EF1Bβ* was fused to the *UidA* reporter gene expressing GUS, and the construct was introduced into Arabidopsis plants. Twelve-day-old seedlings from 10 independent lines showed ubiquitous GUS staining activity throughout the plant (Figure 2A,B,C,D,E) although the expression was relatively low in hypocotyls. GUS activity was most intense in the elongating zones, such as just above the root tips (Figure 2C). Sections from the basal part of the inflorescence stem of 6-week-old plants were used to analyze promoter expression in the vascular elements. GUS activity was mainly concentrated in the vascular bundles, particularly in the xylem and phloem, and in the interfascicular cambium region (Figure 2F). Flower and silique epidermis layers showed fairly strong expression, with the strongest expression in the pistil and stamens (Figure 2G, H), but almost no expression was observed in the seeds (Figure 2I). In summary, GUS expression was highest in elongating cells and in vascular tissues, indicating that EF1Bβ might be associated with cell wall biosynthesis in interfascicular and xylary fibers in addition to its role in translation elongation.

**Figure 3:**
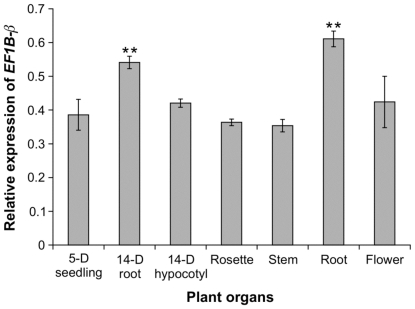
Analysis of *EF1Bβ* expression in different tissues of Arabidopsis, including 5-day-old seedling, hypocotyl and roots from 14-day-old plants, rosette leaves from 4-week-old plants, and inflorescence stem, root and flower from 6-week-old plants. Data represent mean transcript abundance ± SD relative to *EF1α* and *elF4A1* from three independent experiments each replicated three times. ** indicates significant difference relative to stem transcript level at *P*≤0.01.

We also investigated the expression pattern of the *EF1Bβ* gene in different tissues of WT Arabidopsis by qRT-PCR (primers listed in Table S1). *EF1Bβ* transcripts were detected in all of the tissues under study (Figure 3), but transcript abundance varied among the tissues. Relatively higher levels of *EF1Bβ* transcript were detected in root samples from seedlings and 6-week-old plants than in other tissues (*P*≤0.01) (Figure 3). This expression pattern is in accordance with data obtained using the *EF1Bβ* promoter::GUS fusion (Figure 2).

### Genetic analysis and complementation of the *efβ* mutant (SALK_046102C)

**Figure 4:**
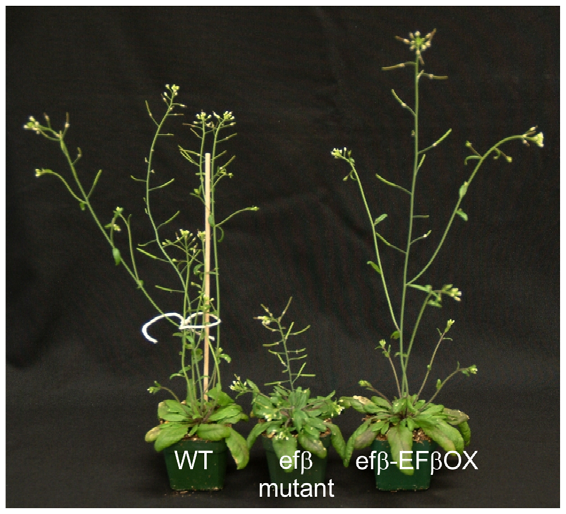
Phenotypes of Arabidopsis wild type, *efβ and* EFβOX-complemented plants. Complemented mutant plant was transformed with a *35S::EF1Bβ cDNA.*

To dissect the role of EF1Bβ in cell wall formation, we initially confirmed the genetic basis and phenotype of *efβ*, a T-DNA insertion mutant of *EF1Bβ* (SALK_046102C). At the seedling stage, all the *efβ* plants grew normally and no visible phenotype differing from the WT phenotype was observed. However, a strong dwarf phenotype (Figure 4) developed later during rosette development and reproductive growth in some plants. To determine the nature of the mutation and T-DNA copy number, homozygous dwarf *efβ* was backcrossed (BC) to the WT (Col 0). All 35 BC F_1_ plants showed WT phenotype and the presence of T-DNA. Of 266 BC F_2_ plants, 200 plants showed a WT phenotype, whereas 66 plants showed a mutant phenotype. This result conforms to a theoretical segregation ratio of 3∶1, indicating a monogenic recessive mutation (*P* ≤ 0.01). We randomly selected 32 plants with WT phenotypes from the BC F_2_ population and used genomic DNA from segregating plants as template for PCR to determine the presence or absence of T-DNA. Of 32 plants, 21 plants had T-DNA insertion, whereas 11 plants did not contain T-DNA. This result supports a theoretical ratio of 2∶1 and also reflects a monogenic recessive mutation caused by T-DNA insertion at a single locus (*P*≤0.01).

**Figure 5:**
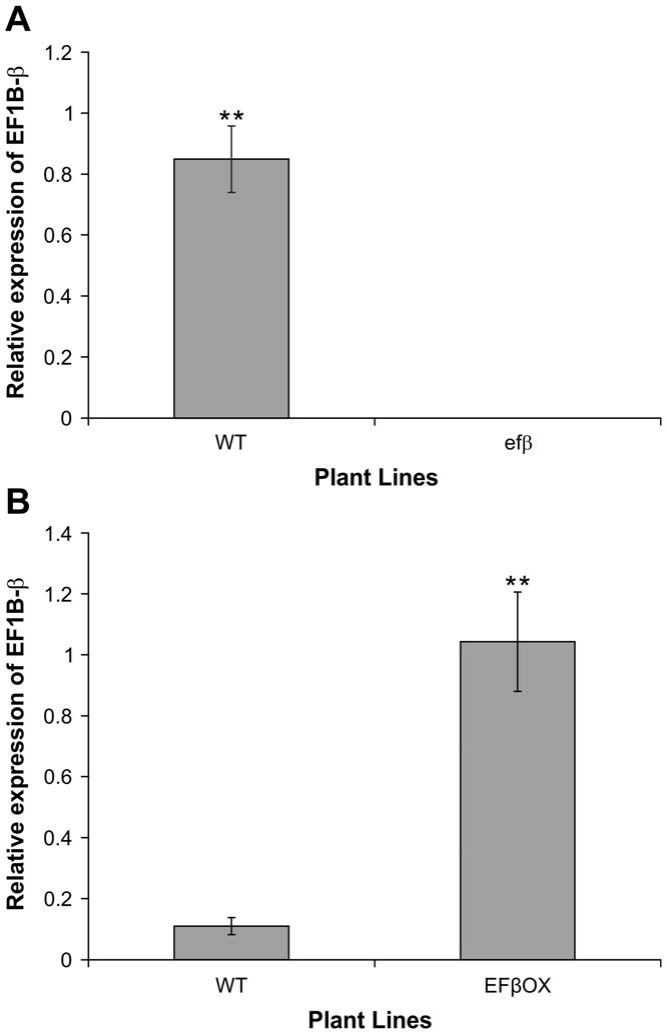
*EF1Bβ* expression in inflorescence stem of WT and *efβ* (A), and of WT and EFβOX (B). Data represent mean transcript abundance ± SD relative to *EF1α* and *elF4A1* from three independent experiments each replicated three times. ** indicates significant difference at *P* ≤ 0.01.

To support the above evidence that disruption of *EF1Bβ* function by T-DNA insertion is responsible for its dwarf phenotype, we performed a complementation experiment, in which mutant *efβ* plants were transformed with an *EF1Bβ* over-expression construct EFβOX. We obtained five independent transformants (T_1_) with a restored (WT) phenotype (Figure 4). In addition, the expression of *EF1Bβ* in WT and *efβ* lines was measured in inflorescence stems from 6-week-old plants by qRT-PCR (primers listed in Table S1). In *efβ*, no expression of *EF1Bβ* was detected, whereas significant expression was detected in the WT (Figure 5A). These results confirm that *efβ* and its stunted phenotype are a direct result of T-DNA insertion into the *EF1Bβ* gene.

### Effect of *EF1Bβ* on cell wall structure

**Figure 6:**
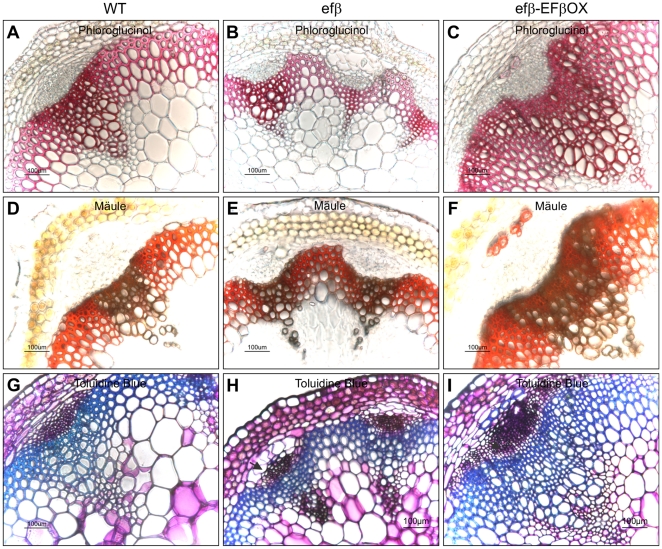
Histochemical analysis of lignin in basal stem cross sections. WT (A,D,G), *efβ* (B,E,H), and EFβOX (C,F,I) plants stained with either phloroglucinol (A–C), Mäule (D-F), or Toluidine Blue staining (G–I). In (H), arrow indicates unusual cell shape in *efβ* stem.

To investigate the consequences of altered *EF1Bβ* expression on cell wall structure, we over-expressed *EF1Bβ* in WT to develop an over-expression line (transformed with *35S::EF1Bβ*, hereafter referred to as EFβOX) and used it in subsequent analysis along with *efβ*. The expression level of *EF1Bβ* in inflorescence stem as determined by qRT-PCR was 10-fold higher in EFβOX compared to WT (Figure 5B). For structural analysis, sections from the basal part of the inflorescence stem of 6-week-old plants of WT, EFβOX and *efβ* were histochemically stained with phloroglucinol, Mäule and Toluidine Blue reagents, and analyzed by light microscopy. Phloroglucinol stain reacts with coniferaldehyde groups in lignin, and the color intensity grossly reflects the total lignin content [29]. With phloroglucinol, *efβ* plants exhibited significantly shrunken interfascicular fibers, and smaller sized and a reduced number of xylem vessels, which suggested that overall lignin content was reduced in *efβ* compared to the WT and EFβOX lines (Figure 6A–C). Yellow-brown coloration from Mäule staining indicated that the xylary elements of all three types of lines were predominantly composed of G-lignin (Figure 6D–F). Red coloration (from Mäule staining) indicated that S-enriched lignin [29] was predominant in the interfascicular region of all three types of lines, and was also present in a patchy pattern within the vascular region of EFβOX (Figure 6F). However, the extent of the red coloration was somewhat lower in *efβ* due to the reduced interfascicular fibers (Figure 6D–F). In Toluidine Blue staining, some cells in between the cortex and phloem of the *efβ* stem showed unusual expanded cell shapes in addition to smaller and reduced xylem elements, and these unusual features were not found in either WT or EFβOX stems (Figure 6G–I). These histochemical observations indicated that disruption of *EF1Bβ* expression caused a reduction in total lignin content (mainly S-lignin) concurrently with growth reduction and a reduction in vascular elements and interfascicular fibers. They also showed that over-expression of *EF1Bβ* increases these elements and fibers in Arabidopsis (Figure 6C, F, I).

### Effect of EF1Bβ on lignin content and composition

**Figure 7:**
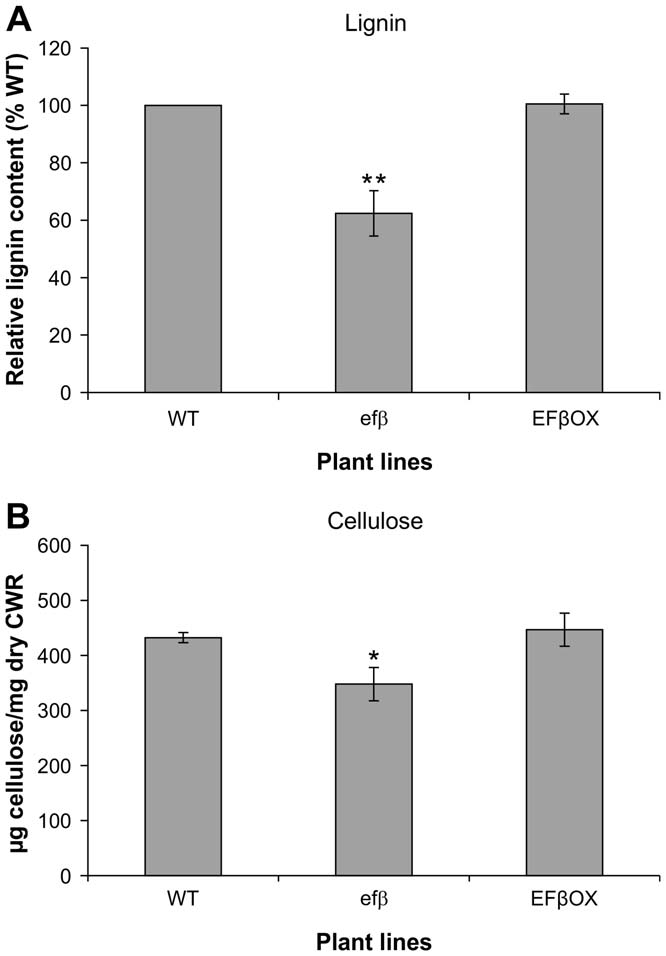
Total lignin and crystalline cellulose in inflorescence stems of EFβOX and *efβ* plants. Lignin is shown relative to that of the WT (A). Cellulose is expressed as µg cellulose mg^−1^ dry CWM (B). Data presented are means ± SD for three independent experiments, each replicated three times. * and ** indicate significant differences at *P*≤0.05 and 0.01, respectively.

Changes in plant phenotype as well as structural and histochemical changes in the inflorescence stem of *efβ* prompted us to investigate the total lignin content in the inflorescence stems of WT, EFβOX and *efβ* plants by the thioglycolic acid method [21]. Total lignin content was reduced by 38% in inflorescence stems of *efβ* plants compared to the WT, whereas the lignin level was almost unaffected in EFβOX plants (Figure 7A). This result is consistent with lignin histochemical analysis (Figure 6).

Angiosperm lignin predominantly contains G and S subunits [10], [22]. Therefore, we determined the relative amounts of G and S monomers in the inflorescence stems by thioacidolysis [30]. This analysis showed that the S-lignin rather than G-lignin (and hence the S/G ratio) was significantly lower in *efβ* plants compared to WT, but no significant differences in S/G ratios could be detected between EFβOX and WT plants (Table 1).

**Table 1:** Lignin composition of *EF1Bβ* gain- and loss-of-function mutants.

Genotype	Monomer composition		S/G
	S lignin (%)	G lignin (%)	
WT	24.56±0.38	75.44±0.38	0.32
EFβOX	22.01±2.51	77.99±2.51	0.28
*efβ*	15.08±2.06*	84.92±2.06*	0.17*

Lignin monomer composition in the inflorescence stem was determined by thioacidolysis method. Data presented as means ± SD of three independent experiments with three technical replicates for each experiment. * indicates significant difference of S/G ratio in *efβ* relative to the WT and EFβOX at *P*≤0.05.

### Effect of EF1Bβ on cellulose content

Cellulose is the main component of cell wall and is indispensible for growth and development. Since disruption of *EF1Bβ* function resulted in a dwarf phenotype and affected lignin, modulation of *EF1Bβ* expression might also affect cellulose content. To investigate this possibility, we measured the cellulose fraction of cell wall from the inflorescence stem using a quantitative colorimetric assay [31]. We found no significant change in cellulose content in EFβOX, whereas *efβ* showed a 20% reduction in cellulose level relative to WT (Figure 7B). A significant reduction (20%) in cellulose level may reflect a general decrease in secondary wall thickening in *efβ* plants.

### *EF1Bβ* over-expression affected cellulose biosynthesis genes

**Figure 8:**
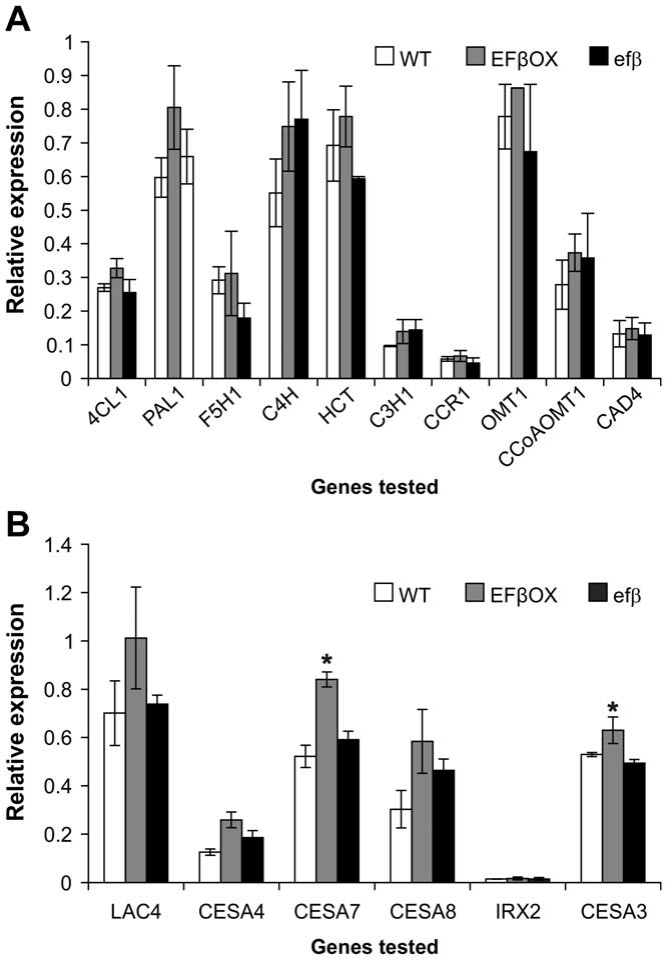
Expression of lignin (A) and cellulose-related (B) genes in WT, EFβOX and *efβ* stems. Data presented as mean transcript abundance ± SD relative to *EF1α* and *elF4A1*of three independent experiments each replicated three times. * indicates significant difference relative to transcript levels in the *efβ* mutant at *P*≤0.05.

Because of the phenotypic and anatomical changes caused by altered *EF1Bβ* expression, we set out to evaluate the effect of *EF1Bβ* on the expression of select cell wall-related genes in inflorescence stems of 6-week-old plants of EFβOX and *efβ* lines. The transcript levels of 10 genes involved in the lignin biosynthetic pathway (listed in [32]) and *LACCASE4* (*LAC4*) did not change significantly between the lines (Figure 8A, B). We also tested the expression pattern of one primary cell wall cellulose synthase gene *CESA3* [33], three secondary cell wall genes *CESA4*, *CESA7* and *CESA8* [34], and a membrane-bound endoglucanase *KORRIGAN1* (*IRX2*). Of all cellulose genes tested, only *CESA3* and *CESA7* showed significant differences in expression, but only in the EFBOX plants, where *CESA3* and *CESA7* expression was increased in relation to expression in *efβ* (Figure 8B).

### *EF1Bβ* rescues the *eli1* phenotype and abolishes ectopic lignification

**Figure 9:**
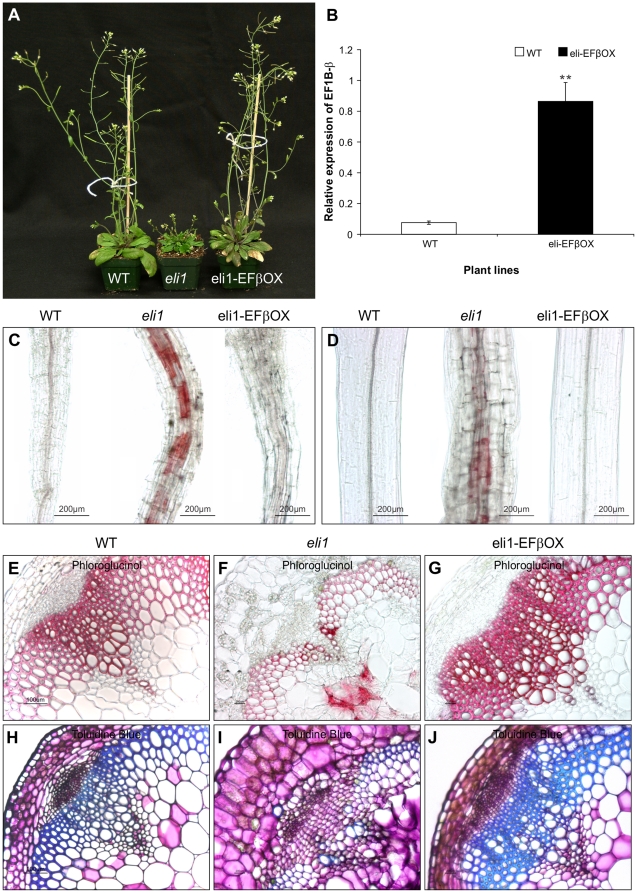
Phenotypes of Arabidopsis wild type, *eli1* and EFβOX-complemented plants (A). Expression of *EF1Bβ* in WT and *eli1*-EFβOX inflorescence stems (B). Data presented as mean transcript abundance ± SD relative to *EF1α* and *elF4A1* of three independent experiments and each replicated three times. ** indicates significant differences relative to WT transcript levels at *P* ≤ 0.01. Phloroglucinol staining of roots (C) and hypocotyls (D) from 5-day-old dark grown seedlings showing ectopic lignification in *eli1* but not in *eli1*-EFBOX or in WT background. Cross sections of the base of the inflorescence stem of 6-week-old plants; WT (E,H), *eli1* (F,I), and *eli1*-EFβOX (G,J) stained with phloroglucinol (E-G) and Toluidine Blue (H-J).

The *eli1* mutant exhibits ectopic lignification and developmental abnormalities, including a stunted phenotype and disorganized xylem [15], due to a mutation in *CESA3* [16]. In addition to cellulose biosynthesis, this gene plays a role in normal cell expansion [15]. By evaluating *eli1* and several other dwarf mutants, these authors found a linkage between cell expansion, the initiation of secondary cell wall formation, and subsequent lignification. As our results suggested a role for EF1Bβ in plant growth and development, we investigated whether EF1Bβ had any role in the *eli1* phenotype. When we expressed *35S::EF1Bβ* in the *eli1* background (hereafter referred to as *eli1*-EFβOX), transformants showed a restored growth phenotype similar to that of the WT (Figure 9A) indicating a role for EF1Bβ in rectifying the growth defects of *eli1*. We also determined the transcript levels of *EF1Bβ* in *eli1*-EFβOX and WT inflorescence stems to establish a link between *eli1* phenotype rescue and *EF1Bβ* transcript level. The rescued plant showed an 11-fold higher expression of *EF1Bβ* relative to WT (Figure 9B). The higher expression of *EF1Bβ* in the restored plants supports the notion that *EF1Bβ* over-expression was responsible for the phenotypic complementation of *eli1*.

Five-day-old seedlings of WT, *eli1* and *eli1*-EFβOX were used to compare lignin accumulation using phloroglucinol-HCl. Seedlings were grown in the dark without sucrose so they remained photosynthetically inactive and avoided any influence of external sugar on lignin [35]. This way the genetic makeup would predominantly contribute to lignin deposition. Under these conditions, phloroglucinol staining in the *eli1* mutant showed patches of cells which accumulated ectopic lignin in both the root and hypocotyl, but were more concentrated in the root (Figure 9C, D). This result is consistent with previous studies on *eli1* [15], [35], which showed strong ectopic lignin accumulation in the roots of seedlings grown in darkness. In contrast, no ectopic lignification was observed in the root or hypocotyl of either WT or *eli1*-EFβOX seedlings grown under the same conditions (Figure 9). These observations indicate that *EF1Bβ* over-expression abolished ectopic lignification in *eli1* and restored the WT phenotype. Basal stem sections of *eli1* showed moderately stained ectopic lignification in the pith parenchyma cells, while the intensity of staining in the xylem elements and interfascicular fibers of *eli1* was relatively low (Figure 9F). Again, this lignin abnormality was absent from the *eli1*-EFβOX plants, showing a restored WT phenotype (Figure 9G).

Sections from the basal part of inflorescence stems were also stained with Toluidine Blue to observe cell structure. Sections of *eli1* exhibited smaller cells with disrupted xylem vessel formation and abnormal development of cortex cells, tracheids, and some pith parenchyma cells (Figure 9I) as reported previously [15]. In contrast, sections from the rescued plant showed normal cell size and vascular development similar to that of the WT (Figure 9J). These observations established that *EF1Bβ* can complement the cell expansion defect of *eli1* and ultimately abolish ectopic lignin accumulation in the *eli1* mutant.

### Expression profile of cell wall genes in *eli1*-EFβOX

In the above experiments we found that *EF1Bβ* over-expression in *eli1* background restored the WT phenotype and abolished ectopic lignification in *eli1* (Figure 9). To extend this analysis to the transcriptional level, we compared the expression pattern of phenylpropanoid and cellulose biosynthesis genes, namely *PAL1*, *C4H*, *CCoAOMT1*, *F5H1*, *OMT1*, *CESA3*, *CESA4*, *CESA7* and *CESA8*, in the WT and *eli1*-EFβOX plants using 2-week-old seedlings grown on plates. Except for *CESA3,* no other gene including *CESA7* showed statistically significant differences in expression in WT and *eli1*-EFβOX lines (Figure S2). *CESA3* expression was almost two-fold higher in *eli1*-EFβOX relative to WT.

## Discussion

Accumulating evidence indicates that components of the translational apparatus have functions in cells beyond their conventional role in protein synthesis [36]. In plants, cytoskeleton and cell wall biosynthetic activities now appear to be closely linked [11], [12], [37]. Due to this close relationship and the fact that EF1B is an actin-binding protein [13], we hypothesized that EF1Bβ may play a role in plant development and cell wall biosynthesis. To investigate this hypothesis, we used gain- and loss-of-function mutants of *EF1Bβ* in a detailed study using molecular, biochemical and histological approaches.

Through a localization study, we found that EF1Bβ is likely localized to the plasma membrane and cytosol. The plasma membrane localization is compatible with a role for EF1Bβ in the synthesis of cell wall components, such as cellulose. Cellulose synthesizing machineries are located in the plasma membrane [9], and two other plasma membrane bound proteins, KORRIGAN1 [38] and KOBITO1 [39], are involved in cellulose synthesis although they are not components of the cellulose synthase proteins. Recently, Gu *et al*. showed that cellulose synthase-interactive protein 1 (CSI1), a plasma membrane localized non-CESA protein is directly involved in cellulose synthesis in the primary cell wall through interaction with CESA isoforms [40]. These findings are in agreement with a possible role for EF1Bβ in cellulose biosynthesis.

*EF1Bβ* promoter::reporter gene expression analysis revealed *EF1Bβ* to be preferentially expressed in developing organs, and in developing fibers and vessels that undergo secondary wall synthesis. Relatively moderate-to-high levels of *EF1Bβ* transcript were detected in all tested organs, which indicate the ubiquitous expression of this gene and underscores the importance of EF1Bβ in plant growth and development. This expression pattern was corroborated when a dwarf phenotype was generated by the disruption of *EF1Bβ* expression (particularly in later stages of development), and is supported by the role EF1B plays in yeast growth [3]. Recently Vain *et al*. reported that a homozygous T-DNA insertion mutant of the gene encoding eukaryotic translation initiation factor 4A (*elF4A*) in *Brachypodium distachyon* exhibited a dwarf phenotype (43-46% of the height of the plant without T-DNA insertion) due to a decrease in both cell number and cell size, and the plants were completely sterile [41]. Both elF4A and EF1Bβ are involved in translation, and silencing of the two genes resulted in dwarf phenotypes though *efβ* plants were fully fertile. A stunted growth phenotype was observed in plants with disrupted expression of different genes involved in cellulose and lignin biosynthesis [9], [10], [16]. Arabidopsis *irregular xylem* 1, 3, and 5 mutants which correspond to mutations in *CESA8*, *CESA7*, and *CESA4* are characterized by collapsed xylem vessels and stems with ∼ 70% lower levels of cellulose compared to wild-type plants [42]-[44]. Our results revealed that disruption of *EF1Bβ* also caused significant reductions in lignin and cellulose levels in cell walls and a change in vascular morphology and structure of the inflorescence stem of the *efβ* mutant. As with the Aspen *PttCel9A1* homolog of *KORRIGAN1* [45], the gain-of-function EFβOX plants showed cellulose and lignin contents similar to WT. Reduction of cell size and changes in cell shape due to disruption of cellulose gene was also observed in the *eli1* mutant and was restored to the WT phenotype in the *eli1*-EFβOX plants.

The reduced cellulose and stunted phenotype with changed cell shape in *efβ* are consistent with reduced growth and misshapen cells found in mutants affecting other plasma membrane-associated proteins, including cellulose biosynthesis and related genes such as *CESA3* [15], *KORRIGAN1* [38] and *KOBITO1* [39], and suggest that *EF1Bβ* may be involved in cellulose biosynthesis. Vascular tissues, either primary or secondary, of higher plants play essential roles in the transport of water, nutrients, and signaling molecules and in physical support [46]. So, the alteration in the size and shape of the vessel elements in *efβ* could impact the transport of nutrients and water to the stem, which could contribute to the reduced stem size of the mutant plants. Disruption of lignin regulatory genes, such as *MYB58* and *MYB63* [47], and lignin structural genes, such as hydroxycinnamyl alcohol dehydrogenase (*CAD*) [48], [49] and hydroxycinnamoyl CoA reductase (*CCR)* also resulted in a wide range of developmental defects, including dwarfism, reduction of cell wall thickness, deformed cell shape, and sterility. EF1Bβ is a plasma membrane and cytosolic protein whereas phenylpropanoid enzymes tend to be cytoplasmic or ER localized [10]. Considering the involvement of this EF1Bβ in translational elongation, a direct role for this protein in lignin biosynthesis is unlikely, but rather its role in lignin biosynthesis may be through maintaining normal plant development.

Lignin monomer composition, when expressed as the syringyl/guaiacyl (S/G) ratio, was altered due to down-regulation of the *EF1Bβ* gene in *efβ* plants relative to WT and EFβOX plants. S-rich lignins are predominantly deposited in the interfasicular fibers of Arabidopsis, whereas cell walls of xylem vessels are rich in guaiacyl lignin [35]. The reduced interfascicular fiber region of *efβ* may have resulted in/from lower S-lignin and a lower S/G ratio in the inflorescence stem of *efβ* relative to WT and EFβOX plants. Recently, Berthet *et al*. demonstrated a strong reduction in lignin content with a substantial increase in S/G ratio in an Arabidopsis *laccase* double mutant (*lac4-2lac17*) with higher saccharification efficiency [29]. In contrast, Sonbol *et al*. reported a positive relationship between lower S/G ratio and increased availability of cell wall polysaccharides in Arabidopsis [50], while Srinivasa Reddy *et al*. demonstrated in alfalfa that the S/G ratio is not necessarily related to cell wall digestibility [51]. These apparent contradictions underline the importance of additional research with *EF1Bβ* to elucidate the relation between lignin composition and cell wall digestibility and to determine its potential use as a tool to engineer plant cell walls for higher digestibility.

Stunted growth and reductions in cellulose and lignin contents in *efβ* plants led us to investigate the effects of modulating *EF1Bβ* transcript levels on the expression of select lignin and cellulose biosynthesis genes. However, no significant differences were observed in levels of transcripts of lignin biosynthesis or cellulose synthase genes except for *CESA3* and *CESA7* in either EFβOX or *efβ* relative to the WT. Histochemical analysis revealed increased vascular development in the EFβOX plants which might be related to the upregulation of *CESA3* or *CESA7* transcript levels. Recently Hématy *et al*. reported that a functional *THESEUS1* (*THE1*), a plasma-membrane-bound receptor-like kinase gene, was required for the dwarf phenotype and ectopic-lignin accumulation in greenhouse-grown cellulose-deficient mutants *eli1-1*, *rsw1-10*, and *pom1-2,* but this gene also did not up-regulate any of the monolignol biosynthetic genes [20]. Since *EF1Bβ* is involved in translation elongation, it is likely that this gene predominantly affects cellulose or lignin biosynthesis at the posttranscriptional level or regulates both of these processes in some indirect fashion.

The inhibition of cellulose synthesis triggers a set of characteristic cellular changes and altered transcript levels for hundreds of genes [20]. The ectopic lignification mutant *eli1* has been extensively studied to elucidate the role of the *CESA3* gene in cellulose biosynthesis, cell expansion, and plant morphology and lignin deposition [16]. We over-expressed *EF1Bβ* in the *eli1* background, which successfully abolished ectopic lignifications and restored the WT phenotype. CESA3 is a cell expansion protein and is important for plant development [15], [16]. Phenotypic and functional rescue of *eli1* by EF1Bβ is consistent with a role for this protein in cell expansion. Gene expression patterns of selected cell wall-related genes in WT and *eli1*-EFβOX were unaltered except for *CESA3* which also reflected the reversal of *eli1* characteristics by EF1Bβ. However, upregulation of the *CESA3* transcript level in *eli1*-EFβOX might be related to the elevation of *EF1Bβ* transcript levels, although the mechanism is unknown. Both genes are involved in cell elongation and it is possible that this common role is responsible for this elevation. Varying degrees of ectopic lignification occur in *eli1* [15], *rsw1* [52], *korrigan1* [53], and *det3* [54], and the degree of ectopic lignification was correlated with the degree of cell expansion. However, we did not observe any ectopic lignification in *efβ* (data not shown). As with the Arabidopsis mutants, *pom-pom* and *cobra,* this implies that reduced cell expansion does not necessarily lead to ectopic lignin accumulation [15], [55] and strongly suggests the involvement of other (or indirect) mechanism(s) or feedback affecting the synthesis of other cell wall components. For example, fluorescent live-cell imaging of CESA6 [56] and CESA3 [57] identified significant intracellular Golgi reservoirs of CESA proteins which did not exclusively coincide with cellulose synthase complex (CSC) assembly. Golgi bodies are known to “pause” on microtubules and affect the excretion of CSCs in Arabidopsis [37]. Hence, intracellular trafficking of CESAs could play a role in the developmental and environmental regulation of cell wall composition. In addition, Caño-Delgado *et al*. showed that reduced cellulose synthesis rather than lignification was responsible for reduced growth of and ectopic lignification in the *eli1* mutant [16]. Indirect effects would explain the role of EF1Bβ in lignin biosynthesis as a consequence of the disruption of cellulose biosynthesis.

In conclusion, *EF1Bβ* is a novel regulator of plant development and plays an important role in cell wall formation. Disruption of its expression negatively impacts plant growth and development as well as vascular tissue development. Over-expression of this gene rescued the cell expansion defects of *eli1* mutant, which further confirms an important role for EF1Bβ in plant development. However, EF1Bβ should be studied in more detail to further unravel the mechanism of cell wall biosynthesis and to confirm whether *efβ* is hypostatic or epistatic relative to *eli1*.

## Supporting Information

Figure S1 Confocal images showing localization of EF1Bβ-YFP (A and D) in Arabidopsis root tip cells plasmolyzed with 0.75 M sorbitol. SynaptoRed (SR) was used as a plasma membrane marker (B and E). Panels C and F are the merged images of the YFP and SR channels. Panels A and C are showing bright yellow circular bodies, possibly the accumulation of EF1Bβ-YFP proteins. Lower panels (D-F) show a close-up of root tip cells. Bars  =  10 µm.
(TIF)

Figure S2 Expression of select cell wall-related genes in WT and *eli1*-EFβOX. Data presented as mean transcript abundance ± SD relative to *EF1α* and *elF4A1* of three independent experiments and each replicated three times. * indicates significant differences relative to WT transcript levels at *P* ≤ 0.05.
(TIF)

Table S1 List of primers used in this study.
(DOC)
