# Supplementary figures and images for: The Translation Elongation Factor eEF-1Bβ1 Is Involved in Cell Wall Biosynthesis and Plant Development in Arabidopsis thaliana
Source: PLoS One. 2012 Jan 17;7(1):e30425. doi: 10.1371/journal.pone.0030425 (PMC3260303; doi:10.1371/journal.pone.0030425)

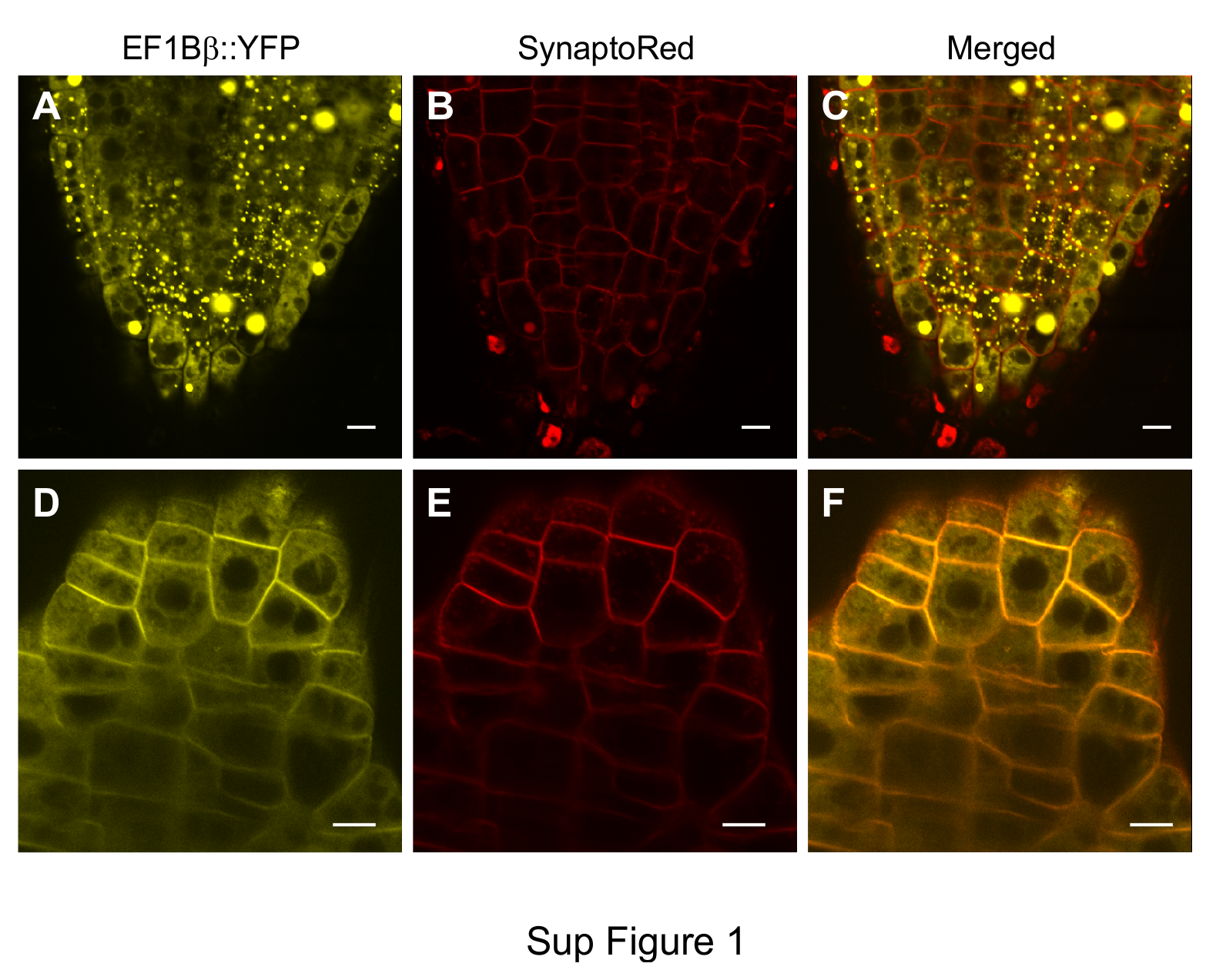

Supplement: Figure S1 — Confocal images showing localization of EF1Bβ-YFP (A and D) in Arabidopsis root tip cells plasmolyzed with 0.75 M sorbitol. SynaptoRed (SR) was used as a plasma membrane marker (B and E). Panels C and F are the merged images of the YFP and SR channels. Panels A and C are showing bright yellow circular bodies, possibly the accumulation of EF1Bβ-YFP proteins. Lower panels (D-F) show a close-up of root tip cells. Bars = 10 µm. (TIF) [file pone.0030425.s001.tif]

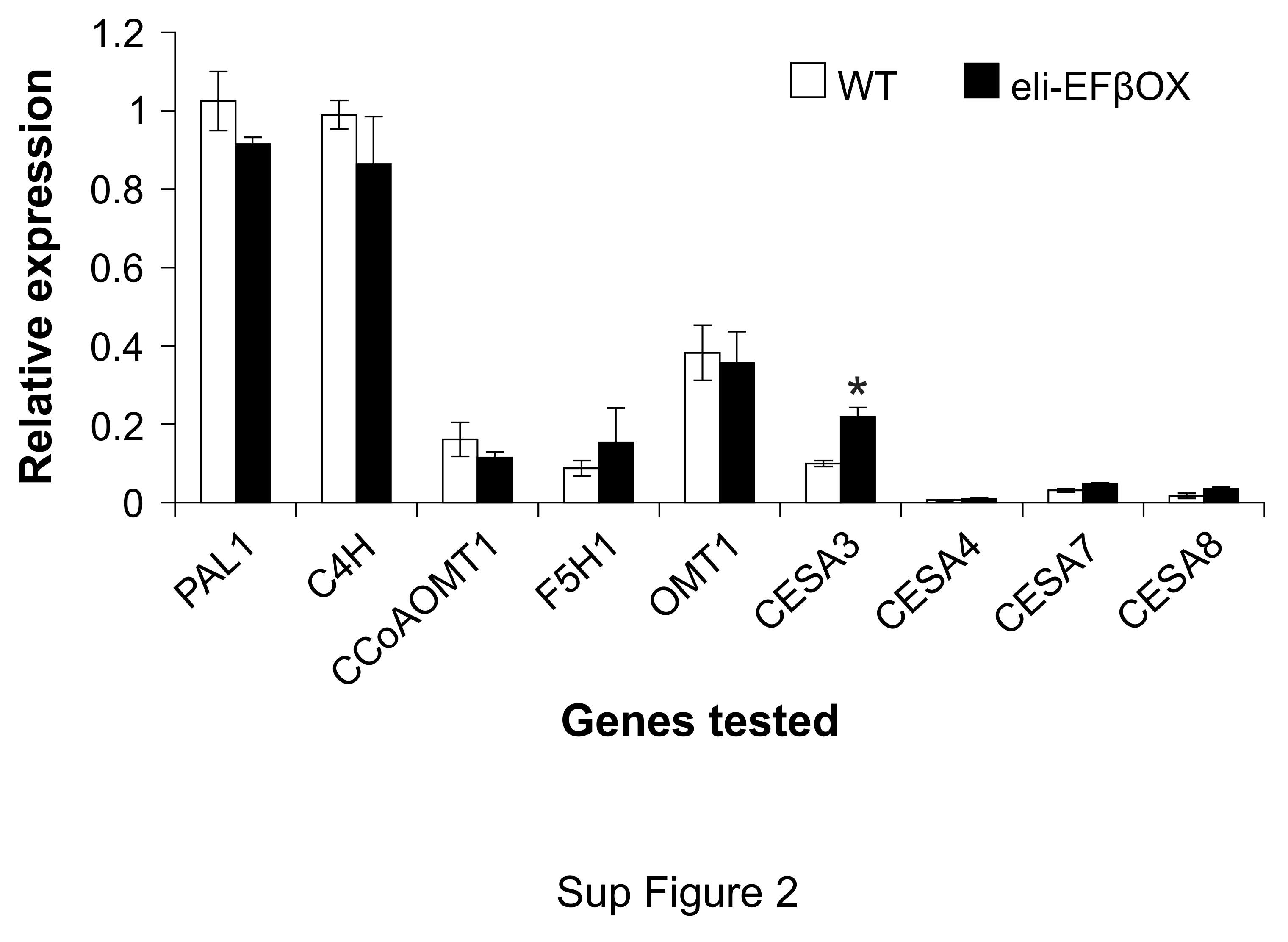

Supplement: Figure S2 — Expression of select cell wall-related genes in WT and eli1-EFβOX. Data presented as mean transcript abundance ± SD relative to EF1α and elF4A1 of three independent experiments and each replicated three times. * indicates significant differences relative to WT transcript levels at P ≤ 0.05. (TIF) [file pone.0030425.s002.tif]
