# Supplementary material for: The Translation Elongation Factor eEF-1Bβ1 Is Involved in Cell Wall Biosynthesis and Plant Development in Arabidopsis thaliana
Source: PLoS One. 2012 Jan 17;7(1):e30425. doi: 10.1371/journal.pone.0030425 (PMC3260303; doi:10.1371/journal.pone.0030425)
Supplement: Table S1 — List of primers used in this study. (DOC) [file pone.0030425.s003.doc]

Table S1 List of primers used in this study

| Primer name | Primer sequence (5′ - 3′) | Purpose | AGI No. | Amplicon size (bp) |
| --- | --- | --- | --- | --- |
| P1 | CAGA**GGATCC**ATGGCAGCATTCCCTAAC | PCR cloning of gene | At1g30230 | 696 |
| P2 | CGTC**GAGCTC**CTACAAAAACTTGGGAAAC |
| P3 | **CACC**TCAGTCAGGATATTTGGAATG | PCR cloning of promoter | At1g30230 | 1934 |
| P4 | CATAGTTTCCGAAGCTCGAACCT |
| P5 | **CACC**ATGGCAGCATTCCCTAACCTTAAC | For YFP fusion | At1g30230 | 693 |
| P6 | TATCTTGTTGAAGGCAACAATGTCACAGC |
| LBb1.3 | ATTTTGCCGATTTCGGAAC | For T-DNA mutant screening |  | 469-769 |
| LP | CAGCCACAGAACCGAAATAAC |
| RP | GTTGACCTTTTCGGAGAGGAG |
| *efβ*_QF | ATGTCCAGAGCTGTGACATTGTTG | qRT-PCR | At1g30230 | 255 |
| *efβ*_QR | CAAGCGATGAACAGAGGGGAAGGAA |
| EFβOX_QF | CGACGAGCATCTTCTCACTCGCA | qRT-PCR | At1g30230 | 159 |
| EFβOX_QR | ACACCAGAGATCCTCAAGAGGGCA |
| PAL1_QF | CCGGACTTCTCACCGGTCGT | qRT-PCR | At2g37040 | 152 |
| PAL1_QR | CGCCGTGCCATTGACTAGCG |
| 4CL1_QF | AGGCTTTGCTCATCGGTCATCCT | qRT-PCR | At1g51680 | 154 |
| 4CL1_QR | ACCTGTTTCGACACGAATTGCTTCA |
| C4H_QF | ACCGGGTGTGCAAGTCACCGA | qRT-PCR | At2g30490 | 140 |
| C4H_QR | AGCCAGCGAGCTTCGCATCA |
| F5H1_QF | CCACGAAACCGCGGAGGACA | qRT-PCR | At4g36220 | 156 |
| F5H1_QR | TCCGGTACGCCCGGTTCCAA |
| HCT_QF | GCAACATCACGCGGCAGATGG | qRT-PCR | At5g48930 | 141 |
| HCT_QR | AAAGCAGGCTGTGGCGGGTC |
| C3H1_QF | TGTCCCGGTGCACAACTTGG | qRT-PCR | At2g40890 | 159 |
| C3H1-QR | CGCTTGCACAGGGGTACG |
| CCR1_QF | CCTCTTCCGACCAAGTGCAAGG | qRT-PCR | At1g15950 | 148 |
| CCR1_QR | GGCCTTTCTCTTGTAAGCTCTTGACTG |
| OMT1_QF | AGCGCGTTCGAGTACCACGG | qRT-PCR | At5g54160 | 159 |
| OMT1_QR | AGCACCAATGCCACCACCAACA |
| CCoAOMT1_QF | TCTCGCCACCGCTCTTGCTC | qRT-PCR | At4g34050 | 141 |
| CCoAOMT1_QR | AGAGCAGGGCCTTCCCTGAAGT |
| CAD4_QF | TCTGGTGGAGGAGGCTGCAACA | qRT-PCR | At3g19450 | 144 |
| CAD4_QR | AGCCAAAGCATTCGTGTTTGAACCA |
| LAC4_QF | CCCTTCCTCCCCCGCAAAACG | qRT-PCR | At2g38080 | 141 |
| LAC4_QR | TTAGCCCAAGGCCGACGGTG |
| CESA3_QF | TCAGAGCGGATGCTTGGTTGGC | qRT-PCR | At5g05170 | 154 |
| CESA3_QR | GGCGTTCAGGTGAGGCAGCA |
| CESA4_QF | CCTCGCCATGGAACCAAACACC | qRT-PCR | At5g44030 | 145 |
| CESA4_QR | AACGCACACGTGACACGC |
| CESA7_QF | CGGCCACAGCGGAGGATTTGA | qRT-PCR | At5g17420 | 142 |
| CESA7_QR | TGTGAGTACGCCTGCCACTCG |
| CESA8_QF | CCCATCTGCAACACTTGTGGTGAA | qRT-PCR | At4g18780 | 158 |
| CESA8_QR | TCGTAAGGATTGCCGCAACGC |
| IRX2_QF | TCGTGCGGCTCTTTCACGTCC | qRT-PCR | At5g49720 | 156 |
| IRX2_QR | AACGCGGCGGCAACAAGAGT |
| UBQ10_QF | GCTCCGACACCATCGACAACG | qRT-PCR | At4g05320 | 160 |
| UBQ10_QR | CTGAGGACCAAGTGGAGGGTGGA |
| EF1a_QF | AACGGTGCCAGTGGGACGTG | qRT-PCR | At5g60390 | 140 |
| EF1a_QR | TGTCACCGGGAAGTGCCTCAA |
| elF4A1_QF | TCAGCCTCTTTCGGATATTCCACCA | qRT-PCR | AT3G13920 | 157 |
| elF4A1_QR | TCCTGCCATGCTGGAGCAAAGT |
| APT1_QF | AGTCATTGATCACGGCTTTTGTCCA | qRT-PCR | At1g27450 | 154 |
| APT1_QR | ACGGTTGCAAGGAACAGCACGA |
| UBC21_QF | AGTCCTGCTTGGACGCTTCAGTCT | qRT-PCR | At5g25760 | 141 |
| UBC21_QR | TTGTGCCATTGAATTGAACCCTCTC |
